# “Is it realistic?” the portrayal of pregnancy and childbirth in the media

**DOI:** 10.1186/s12884-016-0827-x

**Published:** 2016-02-29

**Authors:** Ann Luce, Marilyn Cash, Vanora Hundley, Helen Cheyne, Edwin van Teijlingen, Catherine Angell

**Affiliations:** 1 Faculty of Media & Communication, Bournemouth University, Weymouth House W333, Talbot Campus, Poole, BH12 5BB England UK; 2 Health & Wellbeing Community, Faculty of Health & Social Sciences, Bournemouth University, Bournemouth, UK; 3 Centre for Midwifery, Maternal & Perinatal Health, Faculty of Health & Social Sciences, Bournemouth University, Bournemouth, UK; 4 NMAHP Research Unit, School of Health Science, University of Stirling, Stirling, Scotland UK

**Keywords:** Medical model, Reality television, Labour, Medicalization, Midwifery

## Abstract

**Background:**

Considerable debate surrounds the influence media have on first-time pregnant women. Much of the academic literature discusses the influence of (reality) television, which often portrays birth as risky, dramatic and painful and there is evidence that this has a negative effect on childbirth in society, through the increasing anticipation of negative outcomes. It is suggested that women seek out such programmes to help understand what could happen during the birth because there is a cultural void. However the impact that has on normal birth has not been explored.

**Methods:**

A scoping review relating to the representation of childbirth in the mass media, particularly on television.

**Results:**

Three key themes emerged: (a) medicalisation of childbirth; (b) women using media to learn about childbirth; and (c) birth as a missing everyday life event.

**Conclusion:**

Media appear to influence how women engage with childbirth. The dramatic television portrayal of birth may perpetuate the medicalisation of childbirth, and last, but not least, portrayals of normal birth are often missing in the popular media. Hence midwives need to engage with television producers to improve the representation of midwifery and maternity in the media.

## Background

Considerable debate surrounds the influence media has on people’s perceptions and expectations of birth [1]. A common concern is that reality television (TV) programmes often portray birth as risky, dramatic and painful and that this effects how childbirth is perceived in society [2]. It has been suggested that television portrayals of birth influence decisions made by women (and their families) regarding delivery method (natural birth versus assisted birth including caesarean section), their expectations of the birth (dangerous versus serene), and best place of birth (hospital: considered safe but medicalised versus home considered natural/healthy) [3–9]. It is argued that these influences are in part responsible for the rising rates of interventions in childbirth.

Many high-income countries experience rising rates of childbirth intervention, without much evidence that such interventions lead to improvements in maternal or newborn outcomes [10]. Unnecessary interventions are associated with increased maternal and newborn morbidity. For example, a woman with an uncomplicated pregnancy who opts for a planned caesarean section rather than a vaginal birth is significantly more likely to suffer a cardiac arrest and require a hysterectomy, while her infant is significantly more likely to be admitted to intensive care [11]. Explanatory factors for the rise in interventions and, occasionally, the increase in maternal request for intervention include previous negative birth experiences, and the way that childbirth is portrayed by the media, the latter leading to fear and anxiety about the birth process [12]. Cultural perceptions and societal attitudes are known to influence women’s decisions about when to enter hospital in labour [13, 14]. However, there has been little examination of the relationship between the media, culture and birth-related behaviour.

The literature suggests that many women in the 21^st^ century learn about childbirth through television, as previous generations did, perhaps to a lesser extent, from childbirth manuals [2]. In the United Kingdom (UK), much of this discussion comes in the form of editorials and opinion pieces highlighting the influence of media on mothers’ perceptions of childbirth. These discussions point toward a misrepresentation of normal childbirth on television and in newspapers [15]. Unfortunately, much of this discussion is not underpinned by evidence and is based on a narrative informed by the notion of the ‘media-effects’ tradition, which ’assumes’ that audiences do not critically engage with media messages [16]. Thus women are perceived to be negatively impacted by how childbirth is represented in programmes such as *One Born Every Minute*, *A Baby Story*, *Call the Midwife*, *Pramface Babies*, *Underage and Pregnant* and *16 and Pregnant*. The media-effects theory has long been regarded as too simplistic [17]. Halloran suggested over forty years ago that there is an interaction between the medium and the audience, the latter approaches every media episode with a complicated filter made up not only of their past and present , but also views and hopes for the future [17]*.*

Women are exposed to a number of different viewpoints on and perceptions of childbirth that include: 1) an often stereotypical sensationalised version of the birthing process in the media; 2) stories from friends and relatives; 3) antenatal information provided by midwives, doctors, and other childbirth educators; and 4) personal experiences of giving birth. While we might be critical of women seeking out programmes that depict inaccurate representations of childbirth, we need to remember that media representations are for most women the only opportunity to see a birth [5].

Reality television often presents birth as unpredictable and potentially dangerous, pointing to a steady stream of programmes depicting hypertension, postpartum bleeding, cervical cancer, mothers in preterm labour and diabetes [6]. The media producer needs a ‘hook’ or plot line to engage the viewer. Yet women, often unaware of the range of experiences, continue to watch these programmes as birth preparation, as media users actively seek information and entertainment and select from it to satisfy their needs [18]. It is the impact of using the media to satisfy this need that should be explored in relation to women’s experiences of childbirth.

The paper was designed to determine the gaps in the literature around media and childbirth.

## Methods

Scoping reviews map relevant literature in the field of interest [19], including the ‘grey literature’. The latter includes practitioners' journals, conference papers, unpublished dissertations, books, literature from a range of public, private and voluntary sector bodies and government publications [20]. Scoping reviews do not seek to limit the included literature to a certain type of study (e.g. randomised controlled trials) but instead use broad inclusion criteria [21]. Like a systematic review, the process is rigorous and transparent and documented in detail to enable the review to be replicated [19].

The following electronic databases were searched Medline, Pubmed, NHS Evidence, Academic Search Complete, MIDIRS, Education Research Complete, CINAHL Plus, Scopus, Intute, Zetoc and Web of Knowledge. Databases were searched from their starting date until summer 2014. The following journals were hand-searched, ‘*Midwives (RCM)’, ‘Maternity & Infant Care’, ‘Media, Culture & Society’* and *BMC Pregnancy & Childbirth*.

Databases were searched using the key terms; ‘media representations’, ‘media influence’, ‘media effects’, ‘childbirth’, ‘labour/labor’. ‘The results of searching electronic databases are measured in terms of yield, recall and precision [23]. However, a high yield does not necessarily result in high precision rates, example.g. ‘labour/labor’ yielded many studies on ‘employment’.

A total of 4,014 publications were identified and the titles and abstracts (where available) of the identified publications were screened and included if they met the following inclusion criteria: -

- published in English;
- included research ( qualitative, quantitative or mixed-methods approach);
- contained portrayals of childbirth and/or labour in the media.

**Fig. 1:**
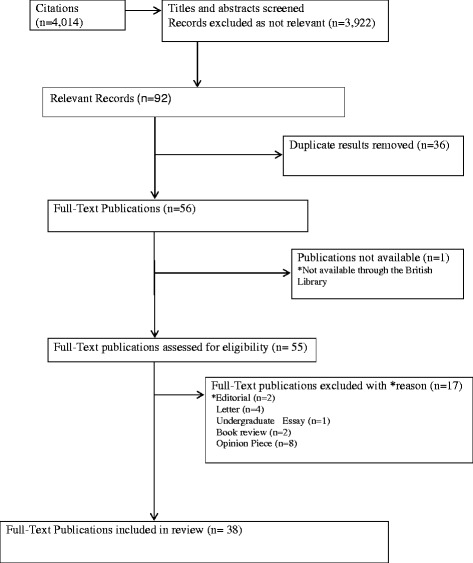
Search and Selection Process

Figure 1 shows 56 publications met the inclusion criteria, as one was not available the full texts of 55 were reviewed, 38 were included and 17 were omitted (2 were editorials, 4 letters, 1 undergraduate essay, 2 book reviews and the remaining 8 were opinion pieces). All papers were read by two authors one with a media background (AL) and one with a health background (MC), thematic analysis was undertaken to identify the key themes through reading and re-reading the included publications. Selected papers were read by the remaining authors to help decide on disagreements between the first two readers and to verify all themes. As this is a secondary analysis of existing literature and no primary data were collected no ethical approval was required.

## Characteristics of publications included in the review

The review comprises twelve qualitative and five quantitative published research studies, three unpublished research pieces and 18 elements of grey literature. The latter includes 13 papers from professional journals, two conference proceedings, two on-line discussion fora and one book chapter. Table 1 lists the included publications, mostly from the US (United States ) (*n* = 17) and the UK (*n* = 10), with four from Canada, three from Australia, and one each from New Zealand and Poland.

**Table 1:** Published research studies in this review

Author (year)	Title	Method	Focus of Study	Place
Declercq *et al.* (2006) [26]	Listening to Mothers II: Report of the Second National U.S. Survey of Women’s Childbearing Experiences.	Quantitative Survey	Experiences and perspectives of childbearing women.	USA
Declercq *et al.* (2013) [50]	Listening to Mothers III: Report of the Third National U.S. Survey of Women’s Childbearing Experiences.	Quantitative Survey	Experiences and perspectives of childbearing women.	USA
Handfield *et al.* (2006) [47]	What do obstetricians think about media influences on their patients?	Quantitative Survey	Australian obstetricians’ perceptions of sources of patient information about birth/ pregnancy, particularly media & Internet.	Australia
Stoll *et al.* (2014) [38]	Why are young Canadians afraid of birth? A survey of childbirth fear and birth preferences among Canadian University Students	Quantitative Survey	Examines attitudes towards birth in young adults who have been socialised into a medicalised birth culture	Canada
Stoll & Hall (2013) [37]	Vicarious Birth Experiences and Childbirth Fear: Does it Matter How Young Canadian Women Learn about Birth?	Quantitative Survey	Explores predictors of childbirth fear for young women	Canada
Clement (1997) [31]	Childbirth on Television.	Qualitative Textual Analysis	Analysis of labour and birth on British television (1993)	UK
Hine (2013) [46]	The Changing Shape Of Pregnancy In New Zealand Women's Magazines: 1970–2008,	Qualitative Content & Textual Analysis	The discursive construction of pregnancy in women’s magazines over 38-year period.	New Zealand
Holdsworth -Taylor (2010) [40]	Portrayals of childbirth: An examination of Internet based Media.	Qualitative Thematic Analysis	Portrayal of childbirth in online media.	Canada
Kline (1997) [4]	Midwife attended births in prime-time television: Craziness, controlling bitches, and ultimate capitulation.	Qualitative Textual Analysis	Portrayal of midwives in television series.	USA
Kline (2010) [24]	Poking Fun at Midwifery on Prime-time Television: The Rhetorical Implications of Burlesque Frames in Humorous Shows	Qualitative Framing Analysis	Assesses rhetorical implications of humorous depictions of midwifery model care in prime-time television.	USA
Longhurst (2009) [48]	YouTube: a new space for birth?	Feminist, post-structuralist geographical perspective	Explores trend of mothers sharing their birthing experiences on You-Tube.	USA
MacLean (2014) [23]	What to expect when you’re expecting? Representations of birth in British Newspapers	Qualitative Content analysis	Newspaper messages of women’s first-person accounts of birth	UK
McIntyre *et al.* (2011) [45]	Shaping public opinion on the issue of childbirth; a critical analysis of articles published in an Australian newspaper	Critical Discourse Analysis	In-depth analysis of childbearing in one single national newspaper	Australia
Morris & McInerney (2010) [6]	Media representations of pregnancy and childbirth: An analysis of reality television programs in the US.	Qualitative Textual Analysis	Analysis of reality-based birth television shows.	USA
Sears & Godderis (2011) [5]	Roar Like a Tiger on TV? Constructions of women and childbirth in reality TV.	Qualitative Thematic analysis	Analysis of ‘Baby Story’ (reality television show).	USA
Song *et al.* (2012) [7]	Women, Pregnancy, and Health Information Online: The Making of Informed Patients and Ideal Mothers.	Qualitative Grounded Theory	Explores how women use Internet to manage (a) their pregnancies & (b) doctor–patient relationships.	USA
Williams & Fahy (2004) [44]	Whose interests are served by the portrayal of childbearing women in popular magazines for women?	Qualitative Textual Analysis	In-depth analysis of childbearing in popular magazine's for women.	Australia

## Results

The key themes were: 1) medicalisation of childbirth, which includes birth being depicted as risky and dangerous and hence something to fear; 2) media the dominant way for women to learn about childbirth, despite the representations being mostly negative; and 3) birth being missing as a normal “everyday” life event. These three themes are presented under the key media groupings of television, print media, new media, and books: old media. The literature revealed a difference in the way that childbirth is depicted in different countries. From the North-American perspective, medicalised childbirth is seen as the only option for mothers-to-be. In the UK, however, this discourse is only starting to emerge [22]. The key frame around childbirth in the UK is that midwives need to engage more with media producers to get accurate representation of childbirth and ensure the uptake of normal birth pathways [23]. Thus, UK media representation of childbirth not only affects a woman’s view on labour, but also that of health care providers.

### Medicalisation of birth

Many of the papers pointed towards a medicalization of birth within the media. As medicine in the US began to gain power and influence in the 20^th^ century *doctors began to displace midwives as the primary provider of maternity care* [24]. This was part of a general trend of the growing prestige of science, which started in the late 19^th^ century in the US. Resulting in the idea that “Giving birth made a woman a mother … a good mother had to learn about mothering from authoritative sources” ([25]: p96). Women have gone along with this medicalised model, remaining relatively passive agents in their own pregnancy; due to the television programmes they are watching and all media more generally [26, 27]. Prior to the 1950s, midwives played a large role in all births; however, when obstetricians began categorising births as either normal or abnormal, their role began to diminish significantly, thus paving the way for the medicalisation of childbirth [4, 5]. ‘Abnormal’ births were situated as potentially difficult, thus requiring a different set of skills that only ‘formally trained and educated doctors’ could perform [5]. Medical intervention in childbirth in the US is now the norm, with nearly half of all births being started artificially, four-fifths of women receiving intravenous fluids, three-quarters receiving epidural analgesia to reduce pain and a third of babies now born by caesarean section [28]. This medicalisation has created disconnect between the pregnant woman and her body. The male medical profession managed to convince middle-class women in the early 20th century to abandon the social model of care as practised by midwives and seek their services in hospitals under the promise of safer and less painful births [7]. Redefining childbirth as pathological helped justify doctors’ authority over the birthing process, legitimised by their specialised knowledge [5]. By medicalising childbirth, the medical establishment rendered both women and midwives as passive agents in the birthing process. The female body, thus, was reduced to an inferior status, and childbirth was now something that was “performed” on a woman, ‘rather than something women performed’ [4, 6–8, 28]. As the mistrust of midwives grew in the US, public opinion about midwives began to change [4], and so did the modes of birth that women were offered [26]. The *Listening to Mothers II* study found that 79% of US births were attended by an obstetrician, with most mothers undergoing technology-intensive care, such as continuous electronic fetal monitoring, intravenous drips, epidurals and/or spinal analgesia, and nearly one-third had had a caesarean section [26].

Although women have several birthing options, the way that reality television constructs birth is contrived as its needs to have entertainment value and hence predominantly promoting a medical model of birth [29]. Murray and Ouellette remind us, that we are aware that reality TV is constructed and partly fictional and still such portrayal whets our desire for the authentic [30]. Similarly, Clement concluded that the images of childbirth viewers see are not an accurate reflection of labour and delivery in Britain [31].

Kitzinger and Kitzinger make the transatlantic link as television has produced a powerful mythology of birth, since a number of television programmes aired in Europe are from North America, and with it the medical model is slowly seeping into the public sphere [32]. Typically on TV doctors deliver babies, whilst in England, midwives are responsible for nearly 57% of all deliveries, rising to nearly 90% for spontaneous deliveries [33].

### Absence of normal birth in the media

What is missing from the public discourse is a conversation about the nature of ‘normal’ birth [34]. What this scoping review has found is that while researchers recognised television programmes as fictional or constructed in a particular way for a viewing audience, they questioned why TV producers present information in this way [5]. Their argument, coming from a ‘behavioural-effects’ stance, is that women make decisions about childbirth based on what they see on these programmes [3, 29, 31, 35, 36] hence the representations need to be more realistic. However, (reality) television is as a genre known to stretch the truth. This medium requires drama, danger, crises and unusual events such as unpredictable and fast deliveries and doctors as heroes, hence a typical birth with a normal slow and lengthy labour without interventions and pain relief and attended by a midwife is less likely to be shown [31].

This stretching of the truth results in a ‘disconnect’ in understanding the media and the role it can play in people’s lives [37, 38]. Some identify a role for midwives in engaging with programme producers and educating them to avoid misrepresentations and allow a more factual portrayal of childbirth and labour [1, 39].

## Television

The literature suggests that many pregnant women find reality television helps them to understand what could happen during childbirth [5, 8, 26, 29, 35]. Reality TV programmes on pregnancy and labour seek to demystify childbirth, and many first-time mothers find it helpful to see inside maternity wards so they know what to expect [40]*.* Holdsworth-Taylor goes one step further adding that Canadian women seek out reality television to add to their knowledge [40], because there is a cultural void [8]. Barker recommends that UK midwives should watch reality television so they can speak to pregnant mothers when they have questions partly based on unrealistic scenarios presented on reality television and soap operas [3]. Haken believes that a woman, who is already disengaged from the medicalised birthing process, is even further removed by watching unrealistic scenes on television [35].

The literature suggests that media portrayal may narrow the options for many women focusing their attention on having a ‘safe’ birth. The media remind us that childbirth is a potentially dangerous condition leaving a woman with no alternative than to ‘choose’ heroic health professionals (mainly doctors) to save them and their babies, and hence accept medical control and interventions [27, 41]. Bak regarded: *‘…these fictional representations of birth act as a filler for the firsthand experience women are denied the opportunity to accumulate. This results in women viewing labour pain as a negative element rather than accepting it as a guide to optimal positioning and a vital element in the physiological feedback that releases additional endorphins and oxytocin, as the body requires’* ([28], p.45)*.*

As women turn to television to learn how others feel and cope with childbirth, birth is no longer a natural experience that women own, rather generations of women have never seen a real-life birth before they themselves experience it [28]. This makes the absence of low-risk undramatic or ‘uneventful’ childbirth on television even more worrying.

## Printed media

What is underreported in the literature is the role newspapers and magazines play in the childbirth experience. Bor found that positive newspaper reports of the first television portrayal of pregnancy in the ‘I Love Lucy’ sitcom in the 1950s, influenced positive audience reception to pregnancy being portrayed on television [42].

Robotham commented anecdotally on UK newspaper headlines such as *The Daily Express’* headline ‘Terror of giving birth in Britain today’, *The Times’* headline, ‘Childbirth is about pain’ and *The Independent’s* story on the ‘conveyor belt of childbirth’ [43]*.* While she did not conduct primary research, she highlighted that such sensationalistic headlines could influence women to avoid seeking a midwife, and instead choose a medicalised birth [43]*.* A recent review of British newspapers highlights a distorted view of birth focusing on risk, which, MacLean argues, prompts a vicious cycle of intervention that starts with fear [23].

Two print-media-based studies were reported in Australia [44, 45]. The first paper studied the role magazines play in expectant mothers’ lives and determined that these are authoritative sources of knowledge and that childbearing is represented in Australian magazines continues the discourse of the medicalisation of childbirth [44]. The second study of one particular national newspaper suggested the general public in Australia may be too worried of the consequences to consider a move away from reliance on traditional medical-led maternity care [45]. Whilst magazines in New Zealand framed pregnancy as an unusual event requiring time, vigilance, and consumption of information, goods and services to successfully perform [46]. More research is needed to look at newspaper and magazine representations of childbirth and the influence they may have.

## New media

Women are increasingly seeking information on the Internet to support/complement what they are watching on television [7, 40, 47, 48]. Literature addressing the Internet’s role in women’s perception of childbirth, however, fails to engage with newer theories of audience reception to help women cope better with what still seems to remain a mystery: *‘regardless of an individual woman’s situation, the experiences of pregnancy and childbirth engender expectations, desires and concerns; thus women seek advice, guidance and care from others with experience and knowledge of the contingencies of these processes’* ([4], p.20). Theroux found that most American women used the Internet at least ten times during their pregnancy, with as most frequent search topic ‘complications of pregnancy’ [49]. The recent *Listening to Mothers III* study revealed that two-in-three pregnant women received regular email updates with information about pregnancy and childbirth [50]. A new finding was that first-time mothers also turned to ‘apps’ for pregnancy and childbirth information—56% rating them as ‘very valuable’ [50]. While US mothers in *Listening to Mothers III* sought out information for themselves, Australia women turned to the Internet for information to discuss with their doctor [47]. US women discussed issues with their doctor’s first, and then turned to the Internet [49]. As yet, there is no study that looks at how British pregnant women engage with the Internet.

Much of the research around usage of the Internet and pregnancy comes from the US. The literature suggests women seek out information on the Internet to get social networking support to have more control over their pregnancy, e.g. *‘… 83% wanted to have more control over decisions affecting their pregnancy and almost two-thirds of women… used the information they obtained from the Internet to help them make decisions about their pregnancy and birth and how their childbirth should be managed’* ([51], p.87).

Women use the Internet to understand what a normal childbirth experience should look like. Song and colleagues note that women have a strong desire for reassurance that what they were experiencing in their pregnancy is ‘normal’ [7]. With the move of birth from the home to hospital, childbirth is missing from everyday life. It has been relegated to something that should be kept from view (unless dramatised within television accounts), as a consequence women have a difficult time in understanding the process of childbirth. Schmid comments that our current lifestyle is too removed from natural experiences [52]. Schmid further notes that: *‘Social messages support the view that birth needs to be medicalised and depersonalized (because of the emphasis only on survival) and these social messages about birth also condition women’s views and beliefs. Therefore interventions in promoting normal birth need to focus on women’s personal lifestyles and wishes as well as on the wider social context of birth and on what needs to be changed in this context to make normal birth possible’* ([52], p.144).

The Internet, more than any other media space creates a cultural ideal for what it means to be a ‘good’ mother. The discourse that emerges online about childbirth is that it is *‘an important event in a woman’s life when she needs to be introspective, focused completely on the labour process and on ensuring her baby’s safe passage into the world’* ([48], p.47). Other research concurs: ‘*…the Internet allows women to educate themselves … it also works to institutionalise a new set of expectations and standards of competence to which women must adhere in order to be considered savvy informed patients and, at the same time, capable mothers’* ([7], p.785).

This moral ideal of a ‘good’ mother dictates that it is important to be seen both online and offline as doing one’s pregnancy ‘right’, and that ‘love, tenderness and care’ are the driving factors [48].

## Books: the old media

Moffat showed how discourses around childbirth and the media that mothers seek out for information have changed over the last 30 years [53]. Books were the main source of information in the 1980s in the US, with television, newspapers and radios being the least used to find out about childbirth. Surprisingly, what has not changed since is the notion of what it means to be a ‘good’ mother, *‘by reaching out for the latest findings and most helpful information available’* ([53], p.67)*.* Even today, many new mothers cited impersonal sources (e.g. books, internet) as their prime source of information about birth [8]. Therefore, women and health professionals should both assess these information resources and together discuss implications for childbirth [2].

## Discussion

Much of the literature in this scoping review fell into a category of media-effects research that suggests that audiences do not critically engage [16]. This ‘hypodermic needle approach’ to media research is based on behavioural-effects theory that tends to rely on a basic understanding of cause-and-effect and assumes that all media audiences are passive. Such studies frequently utilize strongly challenged, if not discredited, theories of direct or causal media effects, which can be problematic because they fail to take into consideration more recent and critical approaches to audience research. One key issue with audience reception is that is not easily observable, except in fragmentary or indirect ways [54]. McQuail also reminds us that audiences are a product of social context and media provision, meaning that an audience can be defined in overlapping ways, and media use reflecting wider patterns of lifestyle, daily routines and time allocation [54]. It would be naive to suggest that women are not influenced by TV programmes; however there are larger, more complicated issues at play in the choices that pregnant women are making. It is this literature that is missing from our body of knowledge and hence the current review.

The research conducted thus far fails to take into consideration that the relationship between cause and effect is not one way. There are many external influences that need to be considered: socioeconomic and environmental factors, fear of childbirth and lack of first-hand knowledge of childbirth. Media representations of childbirth and labour merely reflect the ideologies of society. Ideology refers to an integrated set of frames of reference through which we sees the world and to which all of us adjust our actions [55]. Ideology controls what we see as natural or obvious and colours what we see a particular birth, or a midwifery consultation or our antenatal visit [56]. Temple relying heavily on effects-research argues: *‘different people use the same media in different ways and for different purposes, making it likely that a newspaper will have different effects on different people. People have a well-developed capacity to suppress, forget, distort or misinterpret messages to fit their view of the world’* [57].

### Medical/social model of childbirth

The debates about media portrayal link to the two paradigms of childbirth: the ‘social’ or ‘midwifery’ and the ‘medical’ model [56, 58]. Proponents of the social model adhere to the notion of a physiological labour and a vaginal birth with little or no external intervention [34] as being a normal and therefore a ‘good thing’ in itself; a model traditionally championed by midwives. The medical model, the dominant discourse, encourages women to make use of medical technology, such as monitoring and anesthesia to help reduce the perceived risks and fears associated with giving birth, and in the process move away from labour and birth as physiological processes. Proponents of the medical model argue that childbirth is only safe in retrospect [56], encouraging us to see childbirth as inherently risky for mother and baby. To reduce this perceived risk, a medical birth tends to occur in hospital with electronic fetal monitoring as well as a range of interventions such as forceps or caesarean sections, and typically supervised by a doctor [59].

Media representations often portray technology and interventions as contributing to the medical profession’s success in reducing the risk and uncertainty associated with childbirth [59]. The problem with the promotion of interventions is that there is a paucity of evidence around the routine use of many such childbirth interventions. Leading women to believe that maternity care is designed to ‘manage’ or avert the risks for mother and baby, but often risk management is merely ‘covering’ the hospital/staff in case of litigation [17, 38, 58, 59].

It is important to take into consideration the societal ideological viewpoints of childbirth and labour, for instance, in the US, the predominant approach is the medical model; whilst in the UK both models have currency although the medical model is dominant. Some argue that UK midwives are working in a *‘*blame culture’ that propagates the medical model [57]. Changing this ideology, starting with its portrayal in the media can only be accomplished if midwives engage with popular discourses about the risks and dangers of childbirth that appear on popular reality and fictional television shows. One example of active midwifery input into fictional television is that of Terri Coates, the midwifery advisor on the BBC’s successful television drama *Call the Midwife,* and more recently advisor on a midwifery television drama in Bangladesh [60]*.*

What needs to be taken into consideration is the notion of natural versus medicalised childbirth. Some argue that women prioritise their baby and their own safety, worry about losing control, prefer services that offer, *‘high rates of straightforward birth with guaranteed midwifery support throughout labour and a low need to admit babies to special care baby units’* and want good postnatal and breastfeeding support; thus, suggesting that a medicalised childbirth on television, might not carry over into real life ([61], p. 894). In the US, *‘nonmedicalised representations of pregnancy and birth [on television] would be largely absent and marginalized when they were presented, thereby being hidden from, or distorted in public discourse*’ [6]. This discourse is merely a replication of US social views that having a baby with the aid of a doctor is safer than with a midwife [5, 22]. During the 1990s US midwives tended to be depicted as self- involved, disengaged, unhelpful, and generally mean ‘caregivers’ antagonistic to a woman’s family and friends [4]. Shallow states: *‘… the media has consistently caricatured birth as a horrendous and frightening process that anyone in their right mind would want to avoid at all cost. So who can blame women when terrified, they come to the hospital asking for an elective caesarean section’* [27]*.* Fear surrounding birth, and particularly the fear of birthing outside the ‘safety’ of a hospital, may be responsible for early labour admission and the subsequent cascade of intervention [37, 38, 62].

Handfield *et al*. concur that childbirth in Australia has also been portrayed on television as frightening, overrepresenting deaths and dramatic life-threatening complications [47]. It could be argued that the medical establishment puts forth a medicalised discourse, such as the one that causes fear in women, to maintain power and control over how and where women give birth. Robotham on contacting the BBC after watching particularly concerning scenes on television programmes, *Casualty* and *Holby City,* learnt that there were nurse and medical advisers, but midwifery input was lacking [63]. This reinforces that the discourses surrounding midwifery in the media are not dominant and that seeking out a doctor is the safest way to deliver and prove that one is a ‘good’ mother. Hence midwives must engage more with media producers to ensure normal birth has a place in British-created television programming.

This review has shown that depictions of childbirth and labour indicate that women face social anxieties around their pregnancy. By watching reality television to gain an understanding of what childbirth might be like, viewers must: *‘reflect on ways they themselves must conform to the cultural institutions that surround them. People must submit themselves to the power carried within prescriptions to think and behave in normalized and normalizing ways’* [64], p 194*.*

The most commonly watched shows tend to dramatize pregnancy and birth and over represent obstetric complications and the need for interventions [37]. Women who watch reality TV about childbirth, learn how they should and should not react, i.e. they are socialized into a particular model of childbirth. The latter process is not unique to childbirth, as Kingdon found in the study of representation of depression in the media [65]. Whilst Lupton studying the portrayal of infants in popular media in Australia highlighted this inevitably creates unrealistic expectations of infants in real life [66].

Television can act as a bridge when a life change or transition is occurring, alleviating women’s social anxieties about childbirth [67]. However, fear of birth scores were highest amongst Canadian students who attitudes were shaped by the media [38]. It is clear from our review that women are watching television to learn what to expect during birth, to reassure themselves that they are doing their pregnancy ‘right’.

Underlying all of this is the societal discourse that suggests that it is safer for women to participate in medicalised childbirth, rather than risk a midwife-led birth (in US) or labouring/ birthing at home (in UK). To offset the encroachment of the medicalised model of childbirth, midwives must watch reality and fictional television where childbirth is the focus to be in a position to alleviate fears and answer questions posed by pregnant women. Secondly, midwives and childbirth educators must engage with media producers to create more realistic portrayals of childbirth and labour.

### Strengths and limitations of this research

This review searched and assessed the literature on the effects of the media on childbirth perceptions. The evidence of media influence generally was of low quality. This scoping review only used a selection of the poorly graded evidence to illustrate the key issues. In brief, Consequently many assertions put forward in the academic literature about media influence on perceptions of childbirth are unproven since few studies have measured the impact that such representations have on women and health professionals.

## Conclusion

This review offers insight into current research on the portrayal of childbirth in the media and identifies where gaps exist. It is important to look from an ideological perspective, taking into account issues such as ethnicity, gender and socioeconomic status; none of which were mentioned or considered in the literature included in this scoping review. Future research needs to explore women’s understandings of normal versus risky childbirth, independent of what they watch on television, or what they seek out on the Internet or read in newspapers or magazines. Further research needs to be conducted on where women are getting their information about childbirth and labour. With newer online platforms (Twitter, Facebook, Pinterest, MumsNet) growing rapidly, it is important that researchers understand the role social networking now plays in a woman’s decisions about pregnancy. However as the recent study by Maclean highlighted; UK newspapers have an interest in horror stories and a tendency to suggest that an absence of obstetricians is dangerous, something that she has termed a ‘hierarchy of safety’ [23]. As printed media is still a major part of the mass media, it is imperative that researchers determine if the discourses put forth in the printed press replicate those broadcasted and online. Lastly, it is important to investigate what media producers know about childbirth and labour and their views on the impact that the current representations may be having on women. It is important for midwives to engage with media producers to help improve the representation of childbirth on television, in the same way that midwives should be encouraged to work more with the press [68].
